# Supplementary material for: A novel dimerization site in non-structural protein 5A of hepatitis C virus regulates viral replication fitness
Source: PLoS Pathog. 2026 Aug 5;22(8):e1014239. doi: 10.1371/journal.ppat.1014239 (PMC13472503; doi:10.1371/journal.ppat.1014239)
Supplement: Supplementary file 1 [file ppat.1014239.s006.pdf]

Figure 7A

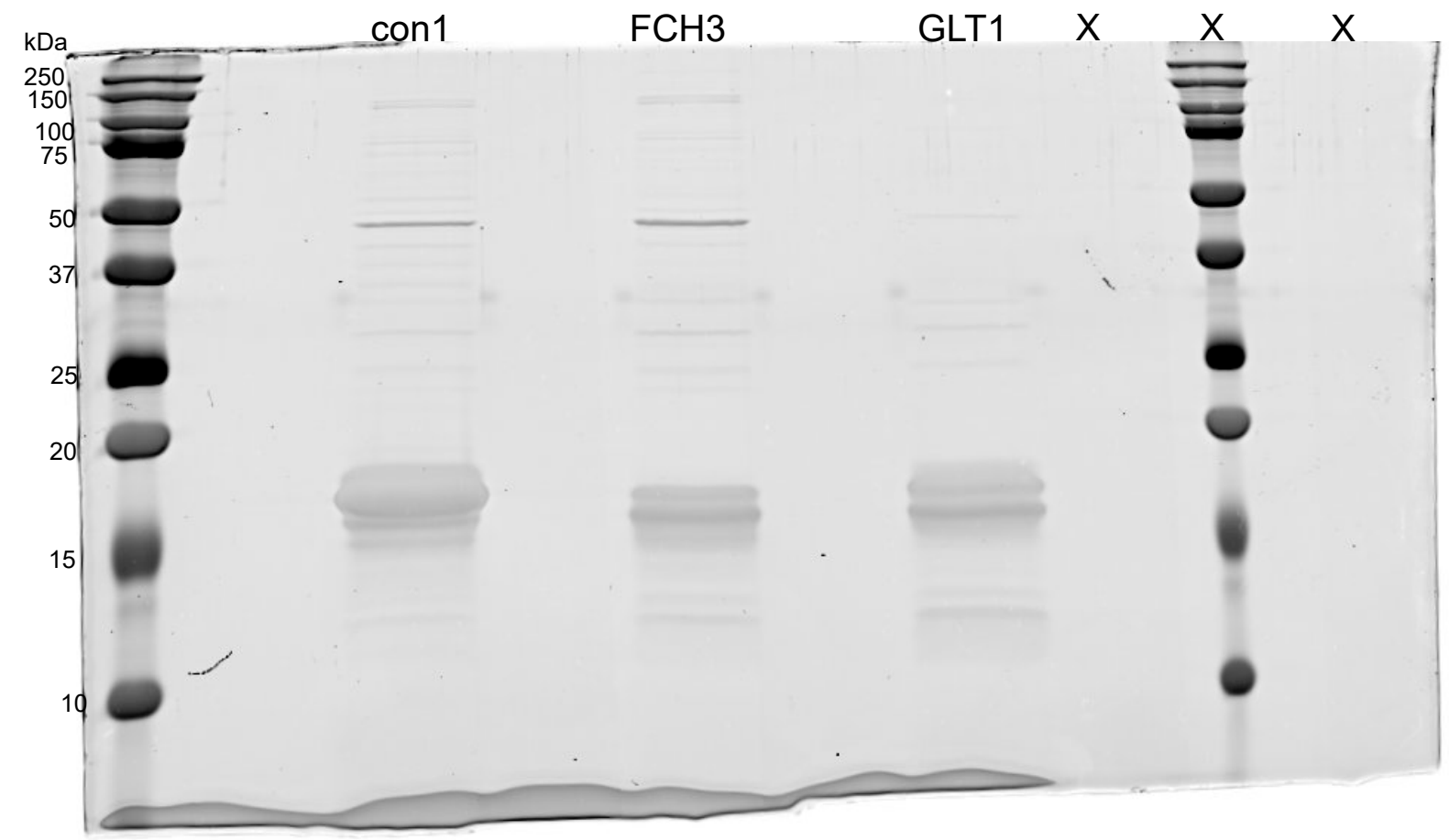

15% Polyacrylamide gel after SDS-PAGE loaded
with 0.8 µg of purified ReED-His
Stained for 30mins in Instant Blue™ and acquired in a
LICORBio Odyssey® M Imaging System using the
700 gel scan (685nm Ex, 720 nm Em) for coomassie blue

Figure S2

NS5A

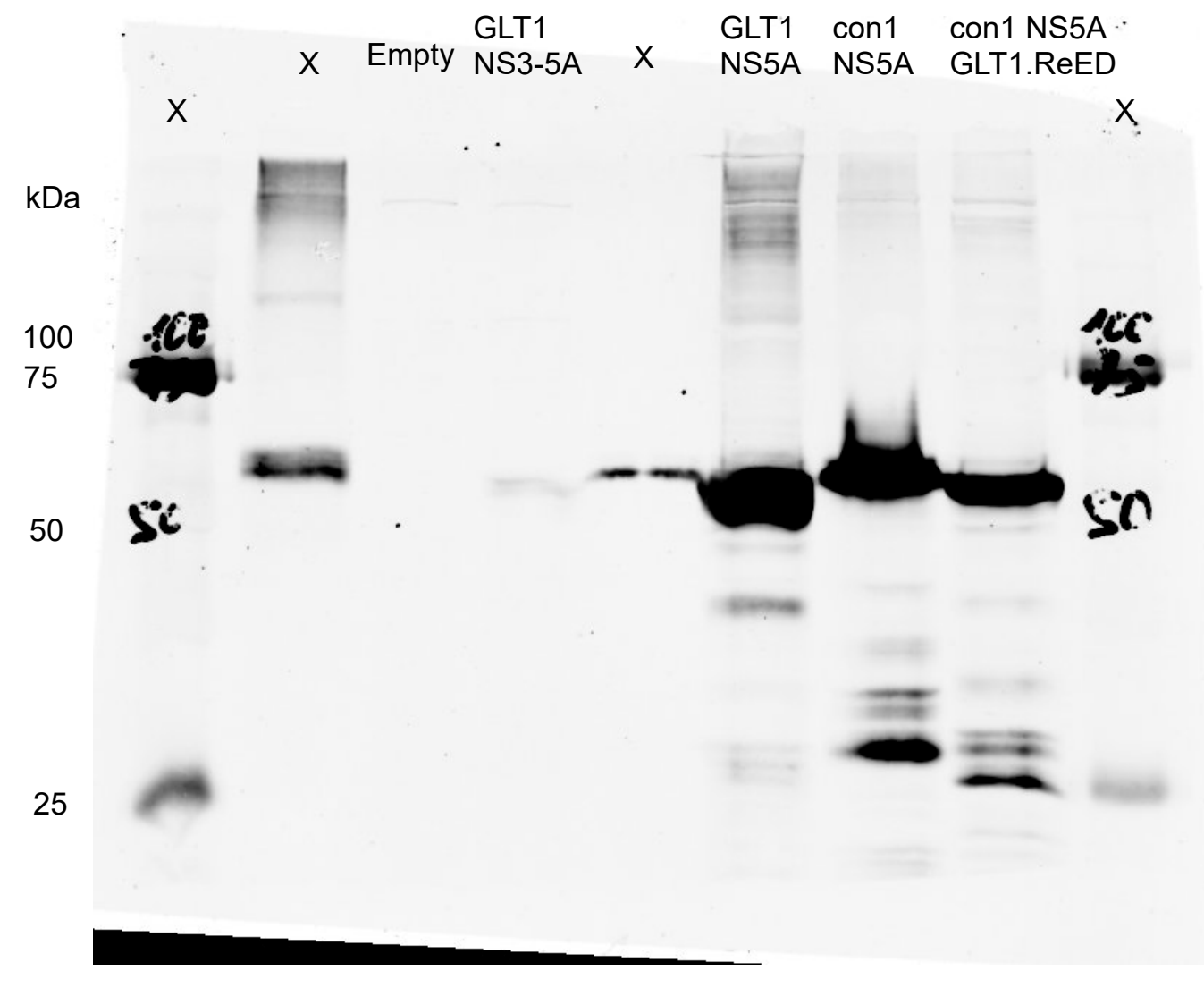

Calnexin

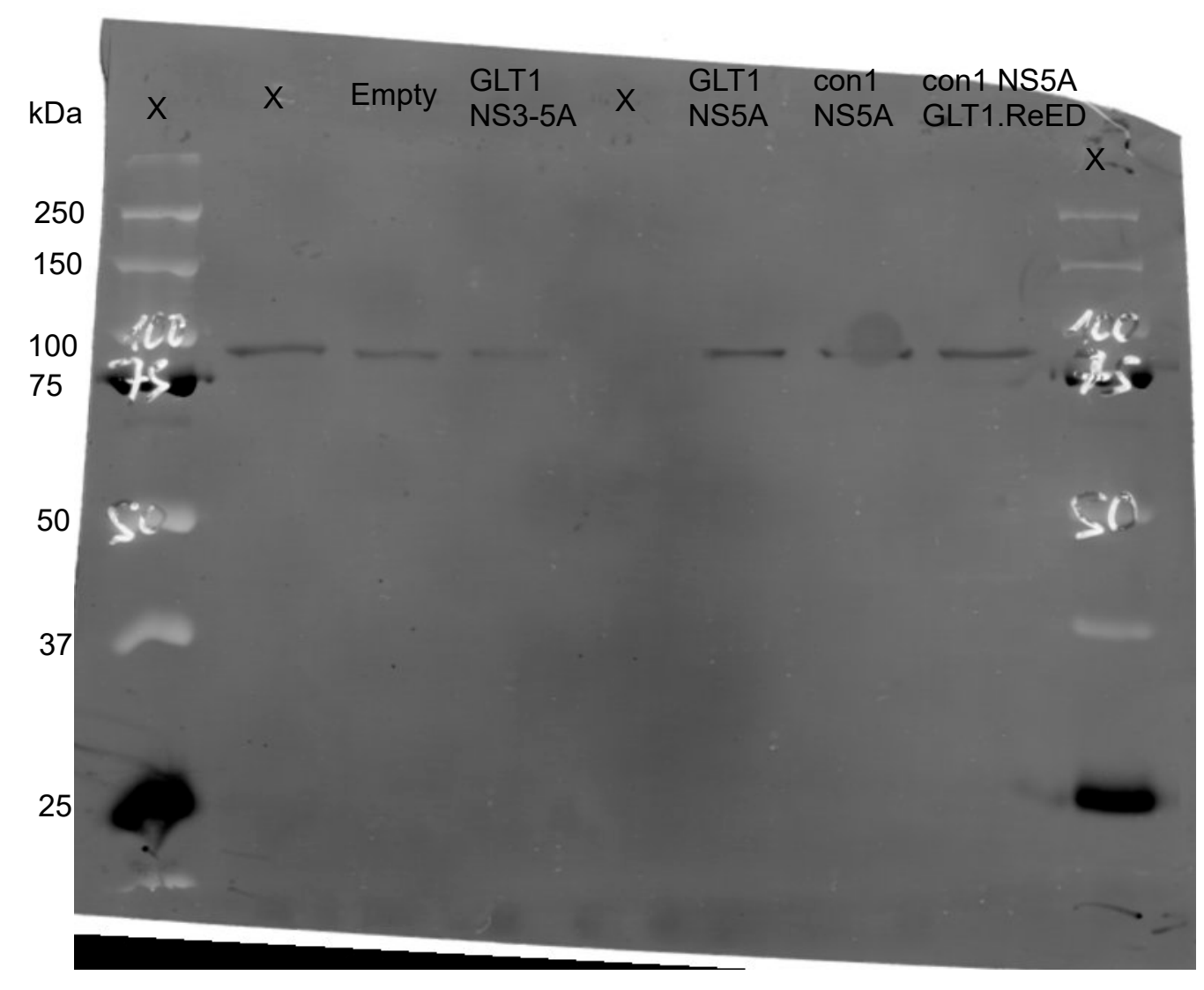

50 µl cell extracts were loaded onto a 10% polyacrylamide gel for SDS-PAGE. Proteins were blotted on a nitrocellulose membrane (Cytiva Amersham™ Protran™
, 0.2 µm pore size) with 400 mA for 2 h in a wet blot system.
The membrane was blocked for 1 h RT in blocking solution (5% skim milk powder in PBS).
The membrane was then incubated with NS5A detecting 9E10 mouse monoclonal antibody (1:5000) and calnexin detecting rabbit polyclonal (Enzo, #ADI-SPA-865-F) (1:1000) in 5% skimmed milk powder in PBS-T (0.1% Tween-20). After three washes in PBS-T the membranes were incubated in
IRDye® 800CW Goat anti-Mouse IgG Secondary Antibody and
VRDye™ 490 Goat anti-Rabbit IgG Secondary Antibody (both 1:5000) in 5% skim milk PBS-T for 1h RT.
After three washes in PBS-T the membranes were imaged in a
LICORBio Odyssey® M Imaging System with the channels 488Ex-530Em (Calnexin) and 785Ex-820Em (NS5A).
