# Supplementary material for: A novel dimerization site in non-structural protein 5A of hepatitis C virus regulates viral replication fitness
Source: PLoS Pathog. 2026 Aug 5;22(8):e1014239. doi: 10.1371/journal.ppat.1014239 (PMC13472503; doi:10.1371/journal.ppat.1014239)
Supplement: Supplementary file 3 [file ppat.1014239.s001.docx]

**S1 Fig: NS5A interaction with known interactors and cyclophilin A inhibitor treatment.** A&B) Detection of indicated proteins via mass spectrometry. WT constructs lacked an HA tag needed for pulldown and thus served as background controls. Data are from four independent biological replicates. Each dot depicts the result of one replicate and the bar indicates the mean of all replicates. X indicates that a factor was not detected in the respective replicate. The dashed grey line indicates the limit of detection (LOD). C) HCV replication assay was performed as in Fig. 1A. 4 h after electroporation, transfected cells were treated with the indicated concentration of the CypA inhibitor Alisporivir. Measurements from 72 h after electroporation were normalized to an untreated control. Data are from three independent biological replicates measured in technical duplicates. IC50 was determined through a nonlinear fit shown as a continuous line. The continuous black line marks cell viability after 72 h normalized to a DMSO control. The results for Con1 and GLT1 not normalized to the untreated control and without IC50 determination as well as the cell viability were already published in (31).
