# Supplementary material for: A novel dimerization site in non-structural protein 5A of hepatitis C virus regulates viral replication fitness
Source: PLoS Pathog. 2026 Aug 5;22(8):e1014239. doi: 10.1371/journal.ppat.1014239 (PMC13472503; doi:10.1371/journal.ppat.1014239)
Supplement: Supplementary file 4 [file ppat.1014239.s002.docx]

**S2 Fig: HCV replication in the context of overexpression of replicase components.** A) Western blot targeting NS5A to confirm its overexpression in the cell lines used in B and Fig. 2D. Calnexin served as a loading control. B) HCV replication assay was performed as shown in Fig. 1A with Con1 GLT1.ReED. Huh7-Lunet SEC14L2 were overexpressing the indicated HCV proteins. Empty refers to a puromycin resistance cassette without additional HCV proteins. Data are from three independent biological replicates measured in technical duplicates. Each dot depicts the result of one replicate, and the bar indicates the mean of all replicates.
