# Supplementary material for: A novel dimerization site in non-structural protein 5A of hepatitis C virus regulates viral replication fitness
Source: PLoS Pathog. 2026 Aug 5;22(8):e1014239. doi: 10.1371/journal.ppat.1014239 (PMC13472503; doi:10.1371/journal.ppat.1014239)
Supplement: Supplementary file 5 [file ppat.1014239.s003.docx]

**S3 Fig: Replication fitness of other gt2a chimeric SGRs.** HCV replication assay was performed with the indicated constructs as shown in Fig. 1A. The gt2a consensus ReED sequence was created based on 15 sequences which were handpicked from HCV GLUE (93) to prevent any bias towards JFH1 or JFH1-derived sequences. JFH1 ∆GDD served as a replication deficient negative control. Data are from three independent biological replicates measured in technical duplicates. Each dot depicts the result of one replicate and the bar indicates the mean of all replicates.
