# Supplementary material for: A novel dimerization site in non-structural protein 5A of hepatitis C virus regulates viral replication fitness
Source: PLoS Pathog. 2026 Aug 5;22(8):e1014239. doi: 10.1371/journal.ppat.1014239 (PMC13472503; doi:10.1371/journal.ppat.1014239)
Supplement: Supplementary file 6 [file ppat.1014239.s004.docx]

**S1 Table: DNA plasmids used and generated during this project.**

| **Name** | **Description** | **GenBank accession** |
| --- | --- | --- |
| pFK i341 NS3-3' Con1 | Bicistronic SGRs of the prototype gt1b wildtype isolates | CAB46677 |
| pFK i341 NS3-3' GLT1 |  | OM222702 |
| pFK i341 NS3-3' Con1 ∆GDD | Replication deficient negative control |  |
| pFK NS3-3' Con1 NS5A-HA | SGRs with an HA-tag in NS5A |  |
| pFK NS3-3' GLT1 NS5A-HA |  |  |
| pFK NS3-3' Con1 GLT1.ReED NS5A-HA |  |  |
| pFK i341 NS3-3' Con1 ∆47 | Adaptive mutation that deletes the whole ISDR and some surrounding  amino acids |  |
| pFK i341 NS3-3' GLT1 ∆47 |  |  |
| pFK i341 NS3-3' Con1 ∆2209-2280 | Deletion of part of the ReED up to the CypA interaction site |  |
| pFK i341 NS3-3' GLT1 ∆2209-2280 |  |  |
| pFK i389 NS3-3' neo GLT1 | GLT1 SGR with a neomycin resistance |  |
| pFK i389 NS3-3' neo GLT1 GND | Replication deficient GLT1 SGR with a neomycin resistance |  |
| pFK i341 NS3-3' Con1 gt1b_cons.ReED | Con1 based chimera harboring the gt1b consensus ReED |  |
| pFK i341 NS3-3' Con1 GLT1.ReED | Con1 based chimera harboring patient derived ReEDs | OM222702 |
| pFK i341 NS3-3' Con1 FCH3.ReED |  | MK092101 |
| pFK i341 NS3-3' Con1 FCH4.ReED |  | MK092103 |
| pFK i341 NS3-3' Con1 NonFCH1.ReED |  | MK092107 |
| pFK i341 NS3-3' Con1 BHCV1.ReED |  | HQ719473 |
| pFK i341 NS3-3' Con1.NS5A-2ACon1.NS5A GLT1.ReED | SGRs with a duplicated NS5A separated by a 2A site for ciscomplementation |  |
| pFK i341 NS3-3' Con1.NS5A  GLT1.ReED-2A-Con1.NS5A  GLT1.ReED |  |  |
| pFK i341 NS3-3' Con1.NS5A-2ACon1.NS5A |  |  |
| pFK i389 NS3-3' JFH1 | Prototype gt2a isolates | AB047639 |
| pFK i389 NS3-3' J6 |  | D00944 |
| pFK i389 NS3-3' JFH1 ∆GDD | Replication deficient negative control |  |
| pFK i389 NS3-3' J6 gt2a.ReED | J6 based chimera harbouring the gt2a consensus ReED |  |
| pFK i389 NS3-3' J6 JFH1.ReED | J6 based chimeras harbouring patient derived ReEDs |  |
| pFK i389 NS3-3' J6 JFH2.ReED |  | AB690456 |
| pFK i389 NS3-3' JFH1 J6.ReED | JFH1 based chimeras harbouring patient derived ReEDs |  |
| pFK i389 NS3-3' JFH1 JFH2.ReED |  |  |
| pFK i389 NS3-3' J6 JFH1.5B3’ | Chimeras between J6 and JFH1 in the context of the J6/JFH1/JFH2 ReED |  |
| pFK i389 NS3-3' J6 JFH1.ReED JFH1.5B3’ |  |  |
| pFK i389 NS3-3' J6 JFH2.ReED JFH1.5B3’ |  |  |

| pFK i389 NS3-3' J6 JFH1.hel |  |  |
| --- | --- | --- |
| pFK i389 NS3-3' J6 JFH1.hel,ReED |  |  |
| pFK i389 NS3-3' J6 JFH1.hel JFH2.ReED |  |  |
| pFK i389 NS3-3' J6 JFH1.3'UTR |  |  |
| pFK i389 NS3-3' J6 JFH1.ReED, 3'UTR |  |  |
| pFK i389 NS3-3' J6 JFH1.3'UTR JFH2.ReED |  |  |
| pFK i389 NS3-3' J6 JFH1.hel, 3'UTR |  |  |
| pFK i389 NS3-3' J6 JFH1.hel,ReED, 3'UTR |  |  |
| pFK i389 NS3-3' J6 JFH1.hel, 3'UTR JFH2.ReED |  |  |
| pFK i389 NS3-3' J6 JFH1.hel, 5B3' |  |  |
| pFK i389 NS3-3' J6 JFH1.hel, R, 5B3' |  |  |
| pFK i389 NS3-3' J6 JFH1.hel, 5B3' JFH2.R |  |  |
| pFK i389 NS3-3' JFH1 J6.ReED J6.5B |  |  |
| pFK i389 NS3-3' JFH1 J6.5B |  |  |
| pFK i389 NS3-3' JFH1 JFH2.ReED J6.5B |  |  |
| pFK i389 NS3-3' JFH1 J6.hel,ReED,5B |  |  |
| pFK i389 NS3-3' JFH1 J6.hel,5B |  |  |
| pFK i389 NS3-3' JFH1 J6.hel,5B JFH2.ReED |  |  |
| pFK i389 NS3-3' JFH1 J6.hel,R,5B3' |  |  |
| pFK i389 NS3-3' JFH1 J6.hel,5B3' |  |  |
| pFK i389 NS3-3' JFH1 J6.hel,5B3' JFH2.R |  |  |
| pFK i389 NS3-3' JFH1 J6.hel,R |  |  |
| pFK i389 NS3-3' JFH1 J6.hel |  |  |
| pFK i389 NS3-3' JFH1 J6.hel JFH2.R |  |  |
| pWPI GLT1 NS3-5A puro | Stable expression of HCV NS proteins or an empty control for  transcomplementation experiments |  |
| pWPI GLT1 NS5A puro |  |  |
| pWPI Con1 NS5A GLT1.ReED puro |  |  |
| pWPI Con1 NS5A puro |  |  |
| pWPI puro |  |  |
| pTM NS3-3' Con1 NS5A-HA | Expression of HCV replicases with an HA-tag in NS5A for  interactome |  |
| pTM NS3-3' GLT1 NS5A-HA |  |  |
| pTM NS3-3' Con1 GLT1.ReED NS5A-HA |  |  |
| pTM eGFP lBit |  |  |
| pTM Con1 NS5A lBit | NS5A tagged with sBit or lBit of NanoLuc for NS5A dimerization assay |  |
| pTM Con1 NS5A GLT1.ReED lBit |  |  |
| pTM Con1 NS5A FCH3.ReED lBit |  |  |
| pTM Con1 NS5A FCH12.ReED lBit |  |  |
| pTM Con1 NS5A Non-FCH1.ReED lBit |  |  |
| pTM Con1 NS5A Non-FCH2.ReED lBit |  |  |
| pTM Con1 NS5A FCH6.ReED lBit |  |  |
| pTM Con1 NS5A FCH7.ReED lBit |  |  |
| pTM Con1 NS5A FCH8.ReED lBit |  |  |
| pTM Con1 NS5A Non-FCH4.ReED lBit |  |  |
| pTM Con1 NS5A Non-FCH5.ReED lBit |  |  |
| pTM Con1 NS5A Non-FCH6.ReED lBit |  |  |
| pTM Con1 NS5A sBit |  |  |
| pTM Con1 NS5A GLT1.ReED sBit |  |  |
| pTM Con1 NS5A FCH3.ReED sBit |  |  |
| pTM Con1 NS5A FCH12.ReED sBit |  |  |
| pTM Con1 NS5A Non-FCH1.ReED sBit |  |  |
| pTM Con1 NS5A Non-FCH2.ReED sBit |  |  |
| pTM Con1 NS5A FCH6.ReED sBit |  | PV083189 |
| pTM Con1 NS5A FCH7.ReED sBit |  | PV083191 |
| pTM Con1 NS5A FCH8.ReED sBit |  | PV083193 |
| pTM Con1 NS5A Non-FCH4.ReED sBit |  | PV083201 |
| pTM Con1 NS5A Non-FCH5.ReED sBit |  | PV083202 |
| pTM Con1 NS5A Non-FCH6.ReED sBit |  | PV083203 |
| pCMV dR 8.91 | Needed for production of lentiviral particles |  |
| pMD2G |  |  |
