# Supplementary material for: A novel dimerization site in non-structural protein 5A of hepatitis C virus regulates viral replication fitness
Source: PLoS Pathog. 2026 Aug 5;22(8):e1014239. doi: 10.1371/journal.ppat.1014239 (PMC13472503; doi:10.1371/journal.ppat.1014239)
Supplement: Supplementary file 7 [file ppat.1014239.s007.docx]

# S1 Method

**Western blot**

50 µl cell extracts were denatured at 95 °C for 10 min and applied to 10% polyacrylamide gels for SDS-PAGE. Proteins were blotted on a nitrocellulose membrane (Cytiva Amersham™ Protran™, 0.2 µm pore size) with constant 400 mA for 2 h with a wet blot system. The membrane was blocked for 1 h in blocking solution (5% skim milk powder in PBS) and incubated overnight at 4 °C with the NS5A detecting 9E10 antibody diluted 1:5,000 in 5% skimmed milk powder in PBS-T (0.1% Tween-20). The membrane was washed three times for 10 min with PBS-T before IRDye® 800CW Goat anti-Mouse IgG Secondary Antibody (LICORbio™) diluted 1:5,000 in blocking solution was added for 1 h at RT. Afterwards, the membrane was washed twice with PBS-T followed by two washes with PBS. Fluorescent signal was detected using the Li-COR Odyssey® M and adjusted for brightness and contrast in Fiji ImageJ (Version 2.16.0/1.54p).

**Cell viability**

Cell viability was measured using the CellTiter-Glo Luminescent Cell Viability Assay (Promega). Cells were seeded in 96-well plates with 1×10^4^ cells/well and incubated in medium supplemented with or without Alisporivir. After 72 h, the samples were measured using the Mithras LB 940 plate reader (Berthold Technologies).
