# Supplementary material for: A novel dimerization site in non-structural protein 5A of hepatitis C virus regulates viral replication fitness
Source: PLoS Pathog. 2026 Aug 5;22(8):e1014239. doi: 10.1371/journal.ppat.1014239 (PMC13472503; doi:10.1371/journal.ppat.1014239)
Supplement: Supplementary file 10006 [file ppat.1014239.s009.pdf]

Point by point response to reviewer comments

Additional Editor Comments (if provided):

Three experts in the field have reviewed the manuscript. Collectively, they are enthusiastic about the work, the importance of further understanding of NS5A, and state that the manuscript is well written. Each reviewer had queries and suggestions that the authors need to address in a revised manuscript. In particular, is the lack of statistics on the graphs.

We highly appreciate the overall very positive perception of our manuscript by all reviewers and editors. We have addressed all queries and questions in the revised manuscript.

We apologize for the lack of statistical analyses. We have added it whenever possible in the revised manuscript for all data n>2.

Part II – Major Issues: Key Experiments Required for Acceptance

Reviewer #1:

None of the graphs include statistical analyses. Differences in replication efficiency are referred to but these should be substantiated by statistical analysis to show significance. This is particularly important given some of the variability shown in some graphs eg Fig 5 B, D.

We apologize for the lack of statistical analyses. We have added it whenever possible in the revised manuscript for all data n>2. For Fig. 5B (now 6B) it was already done in the right panel, Fig. 5D (now 6D) is in the relevant parts only based on two replicates, therefore a statistical analysis would not be meaningful. However, the variation in this particular experiment was very limited and the take-home message from the experiment is in our view rather qualitative than quantitative.

In Fig 2D we are shown complementation by NS5A alone or NS3-5A. However later we are told that the ReED depends on NS5B polymerase. Fig 2D should therefore also include transcomplementation by NS3-5B. This would also help to support the speculative model in Fig 9

Unfortunately, it is in our hands not possible to express detectable levels of NS3-5B in our lentiviral expression system, we tried hard for many years, but failed likely due to packaging capacity issues [1, 2]. However, the replicon based approach shown in Fig. 2A-C formally represents a trans-complementation with NS3-5B, which was not really successful. The fact that already sole expression of NS5A can rescue a low replicator in part, further argues against the need for a more sophisticated trans-complementation approach. However, to address this concern, we have discussed the limitations of our approach in the discussion

section, also pointing to alternative systems in future experiments (e.g. using the Sindbis model established by Brett Lindenbach [3]).

The WB in Fig S2A shows very different levels of expression of NS5A - this makes it difficult to interpret the data in Fig 2D. Very high levels of NS5A appear to be as efficient as low levels of NS3-5A. The authors should at least comment on this. WB of NS3 and NS5B would be helpful to supplement the NS5A blots.

This is indeed hard to understand. However, we do not see, how additional Western-blots on NS3 (only present in NS3-5A) and NS5B (not present in any trans-complemented cell line) would help. The difference in expression level is rather an artifact of the lentiviral expression system (same promoter, differently sized coding units). We have now explicitly pointed at the difference in the revised paper, albeit we cannot offer clear explanations:

"Indeed, overexpression of either GLT1 NS3-5A or GLT1 NS5A increased Con1 replication to a similar extent, compared to a control cell line (Fig. 2D), despite highly divergent expression levels due to the differently sized coding units. This either indicated that the impact achievable by ectopic expression was saturated already at low expression levels, or that the different quality of NS5A expressed in the context of NS3-5A, e.g. due to authentic phosphorylation [1], was more efficient in this experimental design."

Reviewer #2:

No major issues.

Reviewer #3:

Overall the work is well done and controlled. I have two follow up questions.

1) AlphaFold predicts a novel dimerization site within the ReED of NS5A. How conserved is this structural feature? Is it present outside of Gt1b family of NS5As (Gt 2-7)? If it isn't present in all the genotypes, does it provide any insights into replication of these other genotypes?

We are grateful for this excellent suggestion! As requested, we performed modeling of the gt2a ReEDs shown in Fig. 7A and provide this new modeling as Fig. 5C. The modeling shows i) a predicted dimerization site similar to gt1b site in consensus gt2a and J6, albeit predicted with lower confidence ii) no confidently predicted dimerization site in JFH1, in accordance with its insensitivity to ReED regulation iii) a confidently predicted ReED dimerization site, but with different organization than the gt1b one for JFH2. These new results are reported in the results and discussed. This data is fully in line with the experimental evidence on replication and NS5A dimerization. We further analyzed ReED dimerization for the other major genotypes gt3a and gt4a, for which we shown ReED-dependent replication enhancement in our previous study [4] and also found confidently predicted dimerization sites. We could not study other genotypes, since not enough sequence data are available to confidently generate a consensus sequence, which is a prerequisite to count deviations in the ISDR as a signature for potential high replicator variants. Overall, we think that this data fully support our claims and further strengthen the manuscript.

2) The multimeric form of the LCS1D2 proteins in the SEC profiles of Fig 6C is not clear. Could the authors provide an additional method to ascertain the oligomeric state (MALS, DLS, mass photometry etc.)?

To increase the clarity of Figure 6C we added tick marks that indicate the elution volumes of calibration standards corresponding to globular proteins with molecular weights ranging from 6.5 to 75 kDa: Aprotinin (6.5k Da), Ribonuclease A (13.7 kDa), Carbonic anhydrase (29 kDa), Ovalbumin (43 kDa), Conalbumin (75 kDa) [Cytiva Gel Filtration LMW Calibration Kit]. It shows that all three LCS1D2 proteins elute at a volume that would correspond to a 26-28 kDa globular protein. With SEC analysis, the apparent elution volume is influenced much more by hydrodynamic radius (Rh) than by actual molecular weight. This is the reason why IDPs appear larger in SEC. Our SEC analysis shows that the elution volumes of the LCS1-D2 constructs is similar to the one obtained on NS5A-D2 [5], which has been shown to be monomeric when studied by NMR spectroscopy. Thus, the purified LCS1D2 constructs seem to mainly be monomeric. According to our calibration curve, the small SEC peaks observed in the GLT1 and FCH3 ReEDs would correspond to a 51-55 kDa globular protein. Therefore, one could argue for dimeric species.

Providing formal proof of the oligomeric species present in the SEC peaks is not straightforward and is beyond the scope of this study. DLS would provide information on the hydrodynamic radius (Rh) of the proteins, rather than their molecular weight. Given the low molecular weight of the LCS1D2 proteins (~15.5 kDa), mass photometry is not well suited to this application, as the expected signal would be far too weak, particularly at a concentration of ~100nM, which is typically used. SEC-MALS analysis would be one of the methods of choice. However, as we and others have previously demonstrated using SEC, NMR spectroscopy and SAXS, NS5A-D2 (from JFH1 and Con1) is monomeric and the LCS1D2 SEC elution volumes in this study are closely related to those of NS5A-D2. This strongly suggests a monomeric state of LCS1D2 and a low-populated dimeric state for both GLT1 and FCH3 variants [5, 6].

To address this reviewer’s comment, we have included the calibration data to Fig. 2C and explained our conclusions on monomers and dimers better in the results section, as also suggested by both other reviewers.

Part III – Minor Issues: Editorial and Data Presentation Modifications

Reviewer #1:

Page numbers would help the reader.

We apologize and have added page numbers in the revised manuscript.

I would like to see some of the supplemental data moved into the main part of the paper esp Fig S3

We have moved Fig. S3 to the main Fig. 3 as suggested and furthermore converted the entire Fig. S4 (AlphaFold modelling of gt 1b variants, with addition of new data on gt2, 3 and 4) in a

new main Fig. 5. We carefully checked other supplemental data, but felt that it would rather over-convolute the manuscript to transfer more of the supplementary material.

Fig 2E not clear why the last construct with two GLT1 ReEDs is less efficient that the one with one GLT1 ReED?

We have no explanation, but the difference is overall small and not significant. Still, we have rephrased the text, to point clearer to this difference.

Fig 5B - shows that GLT1 dimerisation is marginally higher than Con1, yes in Fig 2D complementation of GLT1 is 1 log higher than Con1. Please explain the discrepancy. What does 'non' refer to?

The differences in the magnitude of effects in the dimerization assay is hard to compare with their impact on replication models, e.g. upon trans-complementation in Fig. 2D. It is even more pronounced in chimeric replicons (Fig. 1B). The 5A-dimerization assay is highly reductionistic. Still, according to our model, dimerization will unleash the stalled replicase, resulting likely in multiple rounds of RNA synthesis, thereby amplifying the magnitude. We have discussed this important point in more detail in the discussion section.

FCH and non-FCH refers to the Barcelona liver transplant cohort, stratifying patients according to the outcome fibrosing cholestatic hepatitis, which was the basis for our previous publication [7]. We have explained the term now in the legend of the revised manuscript.

In Fig 6 A the GLT1 lane does not appear to contain any protein apart from the faint background band. I appreciate that this may express poorly compared to the other two constructs but more needs to be loaded on to the gel to convince. Presumably all constructs were brought to the same concentration for subsequent experiments?

Of course all purified LCS1-D2 proteins were used in the same concentration (fold molar excess) in panel B. GLT1 indeed was more difficult to purify and the gel shown in panel A reflects the differences in elution efficiency. However, we agree that this is misleading and have replaced it with a new gel, adjusted for similar protein loading.

In Fig 6C does the estimated MW of the second peak correspond to a dimer? Proteins of known MW should be analysed as controls to confirm observed MW.

See also response to major comment of Reviewer 3. We added the elution volumes of known MW globular proteins on Fig. 6C., supporting the interpretation of monomers and dimers. However, the apparent MW of intrinsically disordered proteins in SEC is very different from globular proteins.

We have explained the data better in the revised manuscript.

Reviewer #2:

1) Whereas the Introduction covers all the necessary information, I find that the way that several passages are phrased slightly convoluted. To ease understanding, the authors could consider working a bit with the language.

Thanks for bringing our attention to this point. We have rephrased wide parts of the introduction for a clearer structure and hope that it appears less convoluted now.

2) For two reasons, “replication enhancing domain (ReED)” may not be an ideal term: i) the domain is found in different forms in all isolates, and “regulatory” may accordingly be a better term than “enhancing”, and ii) the NS5A structure is defined to include three major domains (I-III), and defining additional domains within this may be confusing. “Region” had perhaps been a better term. Nonetheless, due to the prior publication, I understand if the authors decide to keep the terminology.

We fully agree with every aspect of this comment. When we chose the term ReED in our previous publication, we were not aware of the mechanism, but now we agree that it was not a very wise choice. We initially aimed to term the ReED RFDR (replication fitness determining region), which would have been clearer, but we feared confusion with ISDR. Anyways, since we just set the term in our recent paper, we are afraid to generate even more confusion by again changing it. Still, we have brought up this point in the discussion of the revised manuscript.

3) I would suggest rephrasing of the following statement: “Our data overall suggest that NS5A is a negative regulator of HCV replication fitness with dimerization releasing the inhibitory interaction with helicase and/or polymerase, thereby likely facilitating initiation of RNA synthesis.” The literature and the current study indeed show that NS5A is an important regulator of replication. Stating up front that it is a negative regulator may however give a false impression. It is certainly also a critical positive factor for replication.

Again, we fully agree. The function of the ReED is only one of dozens NS5A has in viral replication and assembly. We have replaced NS5A by “the ReED” in the mentioned statement, toned it down and made further clear, that our model is only tentative and one possibility among others.

4) In Fig. 1, PKRBD is used for PKR binding domain. It might be appropriate to use similar nomenclature for the CypA interaction site.

Sure, we have corrected the scheme according to the suggestion.

5) While the authors state that the insertion of the HA tag into NS5A had no major impact, it does look like it leads to some attenuation across the board. The authors should at least comment on the fact that this could affect any downstream conclusions.

We have mentioned the reduced replication and a statement that it will unlikely affect the interactome, but we agree that this point needs to be clarified.

6) Fig. 4A-D and 7A: Please label residues according to NS5A or the polyprotein for easier interpretation.

Thanks for bringing this up, we have added polyprotein numbering whenever possible. This is indeed helpful for the reader.

7) It is curious that a low concentration pibrentasvir does not affect the NS5A dimerization assay (Fig. 5), but still enhances replication (Fig. 3). The authors argue that inhibition of DI dimerization play a minor role for the

readout of their dimerization assay – but if so, would that not also be expected to play a similar role for dimerization during replication? And by extension, does this not suggest that the ReED have other roles than in dimerization?

We fully agree that this point is somehow puzzling. However, regarding the lack of impact of inhibitors on the dimerization assay, I assume our assumption is correct, given the localization of the split-nanoluc domains and the very low dimerization activity found for the ΔReED construct in Fig. 6B. Additionally, all phenotypic assays on NS5A inhibitors are in the context of the replicase and it has been initially difficult to even show direct binding to purified NS5A, therefore inhibitor binding might be far less efficient in the context of ectopically expressed isolated NS5A.

Anyways, there is no doubt that the ReED also has positive regulatory roles, since deletion still retains replication, but at a low level (see also last point of this reviewer). We have discussed this point in the revised manuscript, also in the context of other reviewer comments.

8) P. 10, end of first paragraph: Spelling mistake: "We are thus led to…"

Although we could not find a spelling mistake, we have changed the term to "This leads to" for higher clarity.

9) The authors argue that "size exclusion chromatography argued for the majority of purified LCS1D2 protein fragments not dimerizing (Fig. 6C)". This figure should be explained better. It is not clear to the non-expert reader how to arrive at the indicated conclusion based on this figure. Is there a control to indicate where expected peaks for monomer and dimers, respectively, would be expected?

See also response to major comment of Reviewer 3. We added the elution volumes of known MW globular proteins on Fig. 6C., supporting the interpretation of monomers and dimers, based on previous analyses. However, the apparent MW of intrinsically disordered proteins in SEC is very different from globular proteins.

We have explained the data better in the revised manuscript.

10) The conclusions drawn from the data in Fig. 6 comes across as disregarding the findings since they don't fit with the hypothesis. Rather, it would make sense to more directly state that the LCSI-DII fragment by itself had a slightly stimulatory effect on NS5B processivity, whereas differences in the ReED did not impact this at 1:1 stoichiometry (would 10-fold or more NS5A:NS5B be expected in vivo?).

This was not our intention. We have modified the statement according to the reviewer's suggestion

11) The data support a role for the NS5A ReED region in regulating replication, and they also convincingly show that the ReED plays a role in regulating NS5A dimerization. It is conceivable that strong ReED interactions stabilize NS5A DI dimers in the corresponding configuration (among several previously proposed dimer configurations). The low dose NS5A inhibitor experiments further suggest that. Although data with genotype 2 strains demonstrate an interplay between the NS5A ReED region, NS3hel and NS5B, any ReED isolate specific direct interaction or regulation of NS3 and/or NS5B is not convincingly demonstrated by the present

manuscript. The proposed model in Fig. 9 must therefore be regarded as speculative, and I recommend that it is removed from the manuscript. One could imagine several other ways how more efficient NS5A dimerization (or change to balance between different dimer or oligomer conformations) could contribute to enhanced RNA replication, and a model like the one schematized here could risk being misleading.

We agree that our model is speculative and that other possible mechanisms are reasonable as well. Still we think, that a visual idea of our hypothesis will be helpful for the reader and we prefer to keep a model in the manuscript.

To address this reviewer comment, we have modified the model, splitting it into a part reflecting the data and one speculative part, with a question mark. We further toned down our statements to make sure, that this is only our view. We further discussed this point, including alternatives, in more depth, also along other reviewer comments.

12) Previous studies showed that the entire ReED could be deleted and even increase replication of a genotype 1b replicon (delta-47; Blight 2000 Science), whereas the same deletion led to complete attenuation in context of an infectious culture system having NS5A from genotype 1b but the other non-structural proteins from JFH1 (Scheel 2012 PLoS Pathog). Furthermore, in the latter system, deletion of the C-terminal part of the ReED was tolerated for genotype 2a (high-replicator JFH1) but not 1a (low replicators). This may further extend to observation of replicon enhancing mutations in LCSI (S2197P/S225P) with opposite effects for infectious cultures systems (Pietschmann 2009 PLoS Pathog., Scheel 2012 PLoS Pathog). The authors could consider to discuss whether findings from the current study explain these previous conundrums.

We did our best to disentangle the literature on this aspect in the revised manuscript. The discrepancy in JFH1 is easy to explain, it is widely independent from the ReED, therefore parts of it can be deleted with little effect. This also explains, why the Bukh lab could literally set any NS5A into his JFH1-virus chimeras. For gt1b it is more complicated, but in line with your previous comments, e.g. #7, the domain deletion data clearly indicate additional, also positive roles of the ReED. The cell culture adaptive effect observed by Blight was not reproducible in our hands, but this was based on selectable replicons, which follow different needs and require far less replication capacity than reporter replicons. The aspect of cell culture adaptive mutations are mainly related to PI4KA, which we have widely addressed in previous studies (mostly in [8]). I am sure that ReED mediated regulation is somehow interwoven, also in the light of the recently identified PI4P binding domain in NS5A D1 [9], but due to the lack of data, we feel that this discussion goes beyond the scope of this paper.

References

1. Reiss S, Harak C, Romero-Brey I, Radujkovic D, Klein R, Ruggieri A, et al. The lipid kinase phosphatidylinositol-4 kinase III alpha regulates the phosphorylation status of hepatitis C virus NS5A. PLoS Pathog. 2013;9(5):e1003359. doi: 10.1371/journal.ppat.1003359 [doi];PPATHOGENS-D-12-02591 [pii].

2. Grunvogel O, Esser-Nobis K, Reustle A, Schult P, Muller B, Metz P, et al. DDX60L Is an Interferon-Stimulated Gene Product Restricting Hepatitis C Virus Replication in Cell Culture. J Virol. 2015;89(20):10548-68. doi: JVI.01297-15 [pii];10.1128/JVI.01297-15 [doi].

3. Kazakov T, Yang F, Ramanathan HN, Kohlway A, Diamond MS, Lindenbach BD. Hepatitis C Virus RNA Replication Depends on Specific Cis- and Trans-Acting Activities of Viral Nonstructural Proteins. PLOS Pathogens. 2015;11(4):e1004817. doi: 10.1371/journal.ppat.1004817.

4. Rothhaar P, Arand T, Seong HG-T, Heuss C, Tulessin M, Wang Z, et al. Highly replicating hepatitis C virus variants emerge in immunosuppressed patients causing severe disease. Nature Communications. 2025;16(1):10948. doi: 10.1038/s41467-025-67174-w.

5. Hanoulle X, Badillo A, Wieruszeski JM, Verdegem D, Landrieu I, Bartenschlager R, et al. Hepatitis C virus NS5A protein is a substrate for the peptidyl-prolyl cis/trans isomerase activity of cyclophilins A and B. J Biol Chem. 2009;284(20):13589-601. doi: M809244200 [pii];10.1074/jbc.M809244200 [doi].

6. Badillo A, Receveur-Brechot V, Sarrazin S, Cantrelle FX, Delolme F, Fogeron ML, et al. Overall Structural Model of NS5A Protein from Hepatitis C Virus and Modulation by Mutations Confering Resistance of Virus Replication to Cyclosporin A. Biochemistry. 2017;56(24):3029-48. doi: 10.1021/acs.biochem.7b00212 [doi].

7. Rothhaar P, Heuss C, Tulessin M, Wang Z, Seong HG, Förster C, et al. Highly replicating hepatitis C virus variants emerge in immunosuppressed patients causing severe disease. Res Sq. 2025. Epub 20250609. doi: 10.21203/rs.3.rs-6194507/v1. PubMed PMID: 40585217; PubMed Central PMCID: PMCPMC12204461.

8. Harak C, Meyrath M, Romero-Brey I, Schenk C, Gondeau C, Schult P, et al. Tuning a cellular lipid kinase activity adapts hepatitis C virus to replication in cell culture. Nat Microbiol. 2016;2:16247. doi: nmicrobiol2016247 [pii];10.1038/nmicrobiol.2016.247 [doi].

9. Bulankina AV, Richter RM, Nettles JH, Yamane D, Grimm C, Karami Y, et al. Phospholipid-driven conformational switching of HCV NS5A links protein folding to replication membrane remodeling. Science Advances. 2026;12(14):eaeb8863. doi: doi:10.1126/sciadv.aeb8863.
