# Supplementary material for: A novel dimerization site in non-structural protein 5A of hepatitis C virus regulates viral replication fitness
Source: PLoS Pathog. 2026 Aug 5;22(8):e1014239. doi: 10.1371/journal.ppat.1014239 (PMC13472503; doi:10.1371/journal.ppat.1014239)
Supplement: Peer review history

# Peer review: Decision Letter 0

**DOI:** 10.1371/journal.ppat.1014239.r001

**Authors:** Alexander Gorbalenya, Richard Kuhn

20 Jun 2026

PPATHOGENS-D-26-01112

A novel dimerization site in non-structural protein 5A of hepatitis C virus regulates viral replication fitness

PLOS Pathogens

Dear Dr. Lohmann,

Thank you for submitting your manuscript to PLOS Pathogens. After careful consideration, we feel that it has merit but does not fully meet PLOS Pathogens's publication criteria as it currently stands. Therefore, we invite you to submit a revised version of the manuscript that addresses the points raised during the review process.

Please submit your revised manuscript by Jul 20 2026 11:59PM. If you will need more time than this to complete your revisions, please reply to this message or contact the journal office at plospathogens@plos.org. When you're ready to submit your revision, log on to https://www.editorialmanager.com/ppathogens/ and select the 'Submissions Needing Revision' folder to locate your manuscript file.

Please include the following items when submitting your revised manuscript:

* A letter that responds to each point raised by the editor and reviewer(s). You should upload this letter as a separate file labeled 'Response to Reviewers'. This file does not need to include responses to any formatting updates and technical items listed in the 'Journal Requirements' section below.

* A marked-up copy of your manuscript that highlights changes made to the original version. You should upload this as a separate file labeled 'Revised Manuscript with Track Changes'.

* An unmarked version of your revised paper without tracked changes. You should upload this as a separate file labeled 'Manuscript'.

If you would like to make changes to your financial disclosure, competing interests statement, or data availability statement, please make these updates within the submission form at the time of resubmission. Guidelines for resubmitting your figure files are available below the reviewer comments at the end of this letter.

As the corresponding author, your ORCID iD is verified in the submission system and will appear in the published article. PLOS supports the use of ORCID, and we encourage all coauthors to register for an ORCID iD and use it as well. Please encourage your coauthors to verify their ORCID iD within the submission system before final acceptance, as unverified ORCID iDs will not appear in the published article. Only the individual author can complete the verification step; PLOS staff cannot verify ORCID iDs on behalf of authors.

We look forward to receiving your revised manuscript.

Kind regards,

Richard J. Kuhn, PhD

Academic Editor

PLOS Pathogens

Alexander Gorbalenya

Section Editor

PLOS Pathogens

Sumita Bhaduri-McIntosh

Editor-in-Chief

PLOS Pathogens

orcid.org/0000-0003-2946-9497

Michael Malim

Editor-in-Chief

PLOS Pathogens

orcid.org/0000-0002-7699-2064

**Additional Editor Comments (if provided):**

Three experts in the field have reviewed the manuscript. Collectively, they are enthusiastic about the work, the importance of further understanding of NS5A, and state that the manuscript is well written. Each reviewer had queries and suggestions that the authors need to address in a revised manuscript. In particular, is the lack of statistics on the graphs.

**Journal Requirements:**

If the reviewer comments include a recommendation to cite specific previously published works, please review and evaluate these publications to determine whether they are relevant and should be cited. There is no requirement to cite these works unless the editor has indicated otherwise.

1) We ask that a manuscript source file is provided at Revision. Please upload your manuscript file as a .doc, .docx, .rtf or .tex. If you are providing a .tex file, please upload it under the item type u2018LaTeX Source Fileu2019 and leave your .pdf version as the item type u2018Manuscriptu2019.

2) Please provide an Author Summary. This should appear in your manuscript between the Abstract (if applicable) and the Introduction, and should be 150-200 words long. The aim should be to make your findings accessible to a wide audience that includes both scientists and non-scientists. Sample summaries can be found on our website under Submission Guidelines:

https://journals.plos.org/plospathogens/s/submission-guidelines#loc-parts-of-a-submission

3) Please upload all main figures as separate Figure files in .tif or .eps format. For more information about how to convert and format your figure files please see our guidelines:

https://journals.plos.org/plospathogens/s/figures

4) Please upload a copy of Figure Fig 1-9 which you refer to in your text on pages 11, 12, , 13, 14, and 15. Or, if the figure is no longer to be included as part of the submission please remove all reference to it within the text.

5) We have noticed that you have uploaded Supporting Information files, but you have not included a list of legends. Please add a full list of legends for your Supporting Information files after the references list.

6) Please amend your detailed Financial Disclosure statement. This is published with the article. It must therefore be completed in full sentences and contain the exact wording you wish to be published.

i) State what role the funders took in the study. If the funders had no role in your study, please state: "The funders had no role in study design, data collection and analysis, decision to publish, or preparation of the manuscript."

ii) If any authors received a salary from any of your funders, please state which authors and which funders..

If you did not receive any funding for this study, please simply state: u201cThe authors received no specific funding for this work.u201d

7) We have amended your Competing Interest statement to comply with journal style. We kindly ask that you double check the statement and let us know if anything is incorrect.

8) PLOS now requires that authors provide the original uncropped and unadjusted images underlying all blot or gel results reported in a submission’s figures or Supporting Information files. This policy and the journal’s other requirements for blot/gel reporting and figure preparation are described in detail at https://journals.plos.org/plosone/s/figures#loc-blot-and-gel-reporting-requirements and https://journals.plos.org/plosone/s/figures#loc-preparing-figures-from-image-files.

When you submit your revised manuscript, please ensure that your figures adhere fully to these guidelines and provide the original underlying images for all blot or gel data reported in your submission. See the following link for instructions on providing the original image data: https://journals.plos.org/plosone/s/figures#loc-original-images-for-blots-and-gels.

In your cover letter, please note whether your blot/gel image data are in Supporting Information or posted at a public data repository, provide the repository URL if relevant, and provide specific details as to which raw blot/gel images, if any, are not available. Email us at customercare@plos.org if you have any questions.

**Reviewers' Comments:**

Reviewer's Responses to Questions

**Part I - Summary**

Please use this section to discuss strengths/weaknesses of study, novelty/significance, general execution and scholarship.

Reviewer #1: This is an interesting study that makes a significant contribution to the confusing area of NS5A function in virus replication. The study is carefully performed with appropriate controls.

Reviewer #2: The current study by Rothhaar et al. presents a detailed analysis of the role of a so-called replication enhancing domain (ReED) spanning the low-complexity region I and domain II of HCV NS5A. It is based on a recent publication by the same authors, where this particular region of NS5A was found to differ specifically among highly replicating isolates from immunosuppressed patients with severe liver disease. The current study builds on this to further mechanistic understanding of this NS5A region and finds that the NS5A ReED from high-replicator strains engage in an additional dimerization interaction leading to enhanced stabilization of a particular NS5A dimer configuration. The study is well executed, with attention to detail, and I only have minor comments to improve the presentation.

Reviewer #3: Despite decades of functional and structural research on the HCV NS5A protein, the functions of this protein remain enigmatic. NS5A appears to be a master coordinator of host and viral proteins, and the major phosphorylated component of the HCV replicase. Despite a lack of enzymatic activity, potent small molecule inhibitors of HCV replication have resistance mutations in NS5A. As a result, these compounds have been classified as NS5A inhibitors. Several of these molecules in combination of more traditional polymerase and protease inhibitors form the basis of modern direct acting antivirals (DAAs) with cure rates >95%.

The manuscript by Rothhaar et al., aims to further our understanding of a interesting replication enhancing domain (ReED) within domain 2 of NS5A. Using AlphaFold the authors suggest that this region forms novel dimerization motif.

**********

**Part II – Major Issues: Key Experiments Required for Acceptance**

Please use this section to detail the key new experiments or modifications of existing experiments that should be absolutely required to validate study conclusions.

Generally, there should be no more than 3 such required experiments or major modifications for a "Major Revision" recommendation. If more than 3 experiments are necessary to validate the study conclusions, then you are encouraged to recommend "Reject".

Reviewer #1: None of the graphs include statistical analyses. Differences in replication efficiency are referred to but these should be substantiated by statistical analysis to show significance. This is particularly important given some of the variability shown in some graphs eg Fig 5 B, D.

In Fig 2D we are shown complementation by NS5A alone or NS3-5A. However later we are told that the ReED depends on NS5B polymerase. Fig 2D should therefore also include transcomplementation by NS3-5B. This would also help to support the speculative model in Fig 9

The WB in Fig S2A shows very different levels of expression of NS5A - this makes it difficult to interpret the data in Fig 2D. Very high levels of NS5A appear to be as efficient as low levels of NS3-5A. The authors should at least comment on this. WB of NS3 and NS5B would be helpful to supplement the NS5A blots.

Reviewer #2: No major issues.

Reviewer #3: Overall the work is well done and controlled. I have two follow up questions.

1) AlphaFold predicts a novel dimerization site within the ReED of NS5A. How conserved is this structural feature? Is it present outside of Gt1b family of NS5As (Gt 2-7)? If it isn't present in all the genotypes, does it provide any insights into replication of these other genotypes?

2) The multimeric form of the LCS1D2 proteins in the SEC profiles of Fig 6C is not clear. Could the authors provide an additional method to ascertain the oligomeric state (MALS, DLS, mass photometry etc.)?

**********

**Part III – Minor Issues: Editorial and Data Presentation Modifications**

Please use this section for editorial suggestions as well as relatively minor modifications of existing data that would enhance clarity.

Reviewer #1: Page numbers would help the reader.

I would like to see some of the supplemental data moved into the main part of the paper esp Fig S3

Fig 2E not clear why the last construct with two GLT1 ReEDs is less efficient that the one with one GLT1 ReED?

Fig 5B - shows that GLT1 dimerisation is marginally higher than Con1, yes in Fig 2D complementation of GLT1 is 1 log higher than Con1. Please explain the discrepancy. What does 'non' refer to?

In Fig 6 A the GLT1 lane does not appear to contain any protein apart from the faint background band. I appreciate that this may express poorly compared to the other two constructs but more needs to be loaded on to the gel to convince. Presumably all constructs were brought to the same concentration for subsequent experiments?

In Fig 6C does the estimated MW ofthe second peak correspond to a dimer? Proteins of known MW should be analysed as controls to confirm observed MW.

Reviewer #2: 1) Whereas the Introduction covers all the necessary information, I find that the way that several passages are phrased slightly convoluted. To ease understanding, the authors could consider working a bit with the language.

2) For two reasons, “replication enhancing domain (ReED)” may not be an ideal term: i) the domain is found in different forms in all isolates, and “regulatory” may accordingly be a better term than “enhancing”, and ii) the NS5A structure is defined to include three major domains (I-III), and defining additional domains within this may be confusing. “Region” had perhaps been a better term. Nonetheless, due to the prior publication, I understand if the authors decide to keep the terminology.

3) I would suggest rephrasing of the following statement: “Our data overall suggest that NS5A is a negative regulator of HCV replication fitness with dimerization releasing the inhibitory interaction with helicase and/or polymerase, thereby likely facilitating initiation of RNA synthesis.” The literature and the current study indeed show that NS5A is an important regulator of replication. Stating up front that it is a negative regulator may however give a false impression. It is certainly also a critical positive factor for replication.

4) In Fig. 1, PKRBD is used for PKR binding domain. It might be appropriate to use similar nomenclature for the CypA interaction site.

5) While the authors state that the insertion of the HA tag into NS5A had no major impact, it does look like it leads to some attenuation across the board. The authors should at least comment on the fact that this could affect any downstream conclusions.

6) Fig. 4A-D and 7A: Please label residues according to NS5A or the polyprotein for easier interpretation.

7) It is curious that a low concentration pibrentasvir does not affect the NS5A dimerization assay (Fig. 5), but still enhances replication (Fig. 3). The authors argue that inhibition of DI dimerization play a minor role for the readout of their dimerization assay – but if so, would that not also be expected to play a similar role for dimerization during replication? And by extension, does this not suggest that the ReED have other roles than in dimerization?

8) P. 10, end of first paragraph: Spelling mistake: “We are thus led to…”

9) The authors argue that “size exclusion chromatography argued for the majority of purified LCS1D2 protein fragments not dimerizing (Fig. 6C)”. This figure should be explained better. It is not clear to the non-expert reader how to arrive at the indicated conclusion based on this figure. Is there a control to indicate where expected peaks for monomer and dimers, respectively, would be expected?

10) The conclusions drawn from the data in Fig. 6 comes across as disregarding the findings since they don’t fit with the hypothesis. Rather, it would make sense to more directly state that the LCSI-DII fragment by itself had a slightly stimulatory effect on NS5B processivity, whereas differences in the ReED did not impact this at 1:1 stoichiometry (would 10-fold or more NS5A:NS5B be expected in vivo?).

11) The data support a role for the NS5A ReED region in regulating replication, and they also convincingly show that the ReED plays a role in regulating NS5A dimerization. It is conceivable that strong ReED interactions stabilize NS5A DI dimers in the corresponding configuration (among several previously proposed dimer configurations). The low dose NS5A inhibitor experiments further suggest that. Although data with genotype 2 strains demonstrate an interplay between the NS5A ReED region, NS3hel and NS5B, any ReED isolate specific direct interaction or regulation of NS3 and/or NS5B is not convincingly demonstrated by the present manuscript. The proposed model in Fig. 9 must therefore be regarded as speculative, and I recommend that it is removed from the manuscript. One could imagine several other ways how more efficient NS5A dimerization (or change to balance between different dimer or oligomer conformations) could contribute to enhanced RNA replication, and a model like the one schematized here could risk being misleading.

12) Previous studies showed that the entire ReED could be deleted and even increase replication of a genotype 1b replicon (delta-47; Blight 2000 Science), whereas the same deletion led to complete attenuation in context of an infectious culture system having NS5A from genotype 1b but the other non-structural proteins from JFH1 (Scheel 2012 PLoS Pathog). Furthermore, in the latter system, deletion of the C-terminal part of the ReED was tolerated for genotype 2a (high-replicator JFH1) but not 1a (low replicators). This may further extend to observation of replicon enhancing mutations in LCSI (S2197P/S225P) with opposite effects for infectious cultures systems (Pietschmann 2009 PLoS Pathog., Scheel 2012 PLoS Pathog). The authors could consider to discuss whether findings from the current study explain these previous conundrums.

Reviewer #3: (No Response)

**********

PLOS authors have the option to publish the peer review history of their article (what does this mean?). If published, this will include your full peer review and any attached files.

If you choose “no”, your identity will remain anonymous but your review may still be made public.

**Do you want your identity to be public for this peer review?**  For information about this choice, including consent withdrawal, please see our Privacy Policy.

Reviewer #1: No

Reviewer #2: No

Reviewer #3: No

[NOTE: If reviewer comments were submitted as an attachment file, they will be attached to this email and accessible via the submission site. Please log into your account, locate the manuscript record, and check for the action link "View Attachments". If this link does not appear, there are no attachment files.]

**Figure resubmission:**

**Reproducibility:**

To enhance the reproducibility of your results, we recommend that authors of applicable studies deposit laboratory protocols in protocols.io, where a protocol can be assigned its own identifier (DOI) such that it can be cited independently in the future. Additionally, PLOS ONE offers an option to publish peer-reviewed clinical study protocols. Read more information on sharing protocols at https://plos.org/protocols?utm_medium=editorial-email&utm_source=authorletters&utm_campaign=protocols

# Author comment: Author response to Decision Letter 1

**DOI:** 10.1371/journal.ppat.1014239.r002

21 Jul 2026

Attachment Submitted filename: ResponsetoReviewers.pdf

# Editor report: Decision Letter 1

**DOI:** 10.1371/journal.ppat.1014239.r003

**Authors:** Alexander Gorbalenya, Richard Kuhn

28 Jul 2026

Dear Prof. Lohmann,

We are pleased to inform you that your manuscript 'A novel dimerization site in non-structural protein 5A of hepatitis C virus regulates viral replication fitness' has been provisionally accepted for publication in PLOS Pathogens.

Before your manuscript can be formally accepted you will need to complete some formatting changes, which you will receive in a follow up email. A member of our team will be in touch with a set of requests.

Please note that your manuscript will not be scheduled for publication until you have made the required changes, so a swift response is appreciated.

IMPORTANT: The editorial review process is now complete. PLOS will only permit corrections to spelling, formatting or significant scientific errors from this point onwards. Requests for major changes, or any which affect the scientific understanding of your work, will cause delays to the publication date of your manuscript.

Should you, your institution's press office or the journal office choose to press release your paper, you will automatically be opted out of early publication. We ask that you notify us now if you or your institution is planning to press release the article. All press must be co-ordinated with PLOS.

Thank you again for supporting Open Access publishing; we are looking forward to publishing your work in PLOS Pathogens.

Best regards,

Richard J. Kuhn, PhD

Academic Editor

PLOS Pathogens

Alexander Gorbalenya

Section Editor

PLOS Pathogens

Sumita Bhaduri-McIntosh

Editor-in-Chief

PLOS Pathogens

orcid.org/0000-0003-2946-9497

Michael Malim

Editor-in-Chief

PLOS Pathogens

orcid.org/0000-0002-7699-2064

***********************************************************

The authors have evaluated and responded to all the previous reviewer queries. Although they were not able to perform all experimental queries, they have revised the manuscript to an acceptable form. The work is an important addition to the HCV field and is now appropriate for PLoS Pathogens.

Reviewer Comments (if any, and for reference):

# Editor report: Acceptance letter

**DOI:** 10.1371/journal.ppat.1014239.r004

**Authors:** Alexander Gorbalenya, Richard Kuhn

Dear Prof. Lohmann,

We are delighted to inform you that your manuscript, "A novel dimerization site in non-structural protein 5A of hepatitis C virus regulates viral replication fitness," has been formally accepted for publication in PLOS Pathogens.

We have now passed your article onto the PLOS Production Department who will complete the rest of the pre-publication process. All authors will receive a confirmation email upon publication.

The corresponding author will soon be receiving a typeset proof for review, to ensure errors have not been introduced during production. Please review the PDF proof of your manuscript carefully, as this is the last chance to correct any scientific or type-setting errors. Please note that major changes, or those which affect the scientific understanding of the work, will likely cause delays to the publication date of your manuscript. Note: Proofs for Front Matter articles (Pearls, Reviews, Opinions, etc...) are generated on a different schedule and may not be made available as quickly.

Soon after your final files are uploaded, the early version of your manuscript, if you opted to have an early version of your article, will be published online. The date of the early version will be your article's publication date. The final article will be published to the same URL, and all versions of the paper will be accessible to readers.

For Research Articles, you will receive an invoice from PLOS for your publication fee after your manuscript has reached the completed accept phase. If you receive an email requesting payment before acceptance or for any other service, this may be a phishing scheme. Learn how to identify phishing emails and protect your accounts at https://explore.plos.org/phishing.

Thank you again for supporting open-access publishing; we are looking forward to publishing your work in PLOS Pathogens.

Best regards,

Sumita Bhaduri-McIntosh

Editor-in-Chief

PLOS Pathogens

orcid.org/0000-0003-2946-9497

Michael Malim

Editor-in-Chief

PLOS Pathogens

orcid.org/0000-0002-7699-2064
